# Impact of an Online Discussion Forum on Self-Guided Internet-Delivered Cognitive Behavioral Therapy for Public Safety Personnel: Randomized Trial

**DOI:** 10.2196/59699

**Published:** 2024-08-14

**Authors:** Hugh C McCall, Heather D Hadjistavropoulos

**Affiliations:** 1 Department of Psychology University of Regina Regina, SK Canada; 2 PSPNET University of Regina Regina, SK Canada

**Keywords:** internet, cognitive behavioral therapy, forum, persuasive design, generalized anxiety disorder, major depressive disorder, posttraumatic stress disorder, public safety personnel

## Abstract

**Background:**

Internet-delivered cognitive behavioral therapy (ICBT) is an effective and accessible treatment for various mental health concerns. ICBT has shown promising treatment outcomes among public safety personnel (PSP), who experience high rates of mental health problems and face barriers to accessing other mental health services. Client engagement and clinical outcomes are better in ICBT with therapist guidance, but ICBT is easier to implement on a large scale when it is self-guided. Therefore, it is important to identify strategies to improve outcomes and engagement in self-guided ICBT and other self-guided digital mental health interventions. One such strategy is the use of online discussion forums to provide ICBT clients with opportunities for mutual social support. Self-guided interventions accompanied by online discussion forums have shown excellent treatment outcomes, but there is a need for research experimentally testing the impact of online discussion forums in ICBT.

**Objective:**

We aimed to evaluate a transdiagnostic, self-guided ICBT intervention tailored specifically for PSP (which had not previously been assessed), assess the impact of adding a therapist-moderated online discussion forum on outcomes, and analyze participants’ feedback to inform future research and implementation efforts.

**Methods:**

In this randomized trial, we randomly assigned participating PSP (N=107) to access an 8-week transdiagnostic, self-guided ICBT course with or without a built-in online discussion forum. Enrollment and participation were entirely web-based. We assessed changes in depression, anxiety, and posttraumatic stress as well as several secondary outcome measures (eg, treatment engagement and satisfaction) using questionnaires at the pre-enrollment, 8-week postenrollment, and 20-week postenrollment time points. Mixed methods analyses included multilevel modeling and qualitative content analysis.

**Results:**

Participants engaged minimally with the forum, creating 9 posts. There were no differences in treatment outcomes between participants who were randomly assigned to access the forum (56/107, 52.3%) and those who were not (51/107, 47.7%). Across conditions, participants who reported clinically significant symptoms during enrollment showed large and statistically significant reductions in symptoms (*P*<.05 and *d*>0.97 in all cases). Participants also showed good treatment engagement and satisfaction, with 43% (46/107) of participants fully completing the intervention during the course of the study and 96% (79/82) indicating that the intervention was worth their time.

**Conclusions:**

Previous research has shown excellent clinical outcomes for self-guided ICBT accompanied by discussion forums and good engagement with those forums. Although clinical outcomes in our study were excellent across conditions, engagement with the forum was poor, in contrast to previous research. We discuss several possible interpretations of this finding (eg, related to the population under study or the design of the forum). Our findings highlight a need for more research evaluating the impact of online discussion forums and other strategies for improving outcomes and engagement in self-guided ICBT and other digital mental health interventions.

**Trial Registration:**

ClinicalTrials.gov NCT05145582; https://clinicaltrials.gov/study/NCT05145582

## Introduction

### Background

Internet-delivered cognitive behavioral therapy (ICBT) is a psychological treatment in which clients learn evidence-based cognitive behavioral therapy treatment strategies via web-based modules, often with therapist guidance via email or phone. Hundreds of randomized trials have demonstrated that ICBT is similarly effective to face-to-face psychotherapies for treating depression and anxiety [1]. There are 2 key advantages of ICBT and other digital mental health interventions (DMHIs): the ability of clients to access them privately and conveniently at practically any time and location [2-4] and the tendency for DMHIs to require less therapist time per client than traditional face-to-face psychotherapies [2,4].

ICBT is sometimes offered in a purely self-guided format (ie, without a therapist). Meta-analyses have shown that self-guided ICBT is at least slightly less effective than therapist-guided ICBT; this assertion is based on differences in effect sizes observed in separate meta-analyses of guided [1] and self-guided [5,6] ICBT, meta-analyses including subgroup analyses of both guided and self-guided ICBT [7-9], a meta-regression in which human contact predicted more favorable ICBT outcomes [10], a meta-analysis of randomized trials directly comparing guided and self-guided ICBT [11], and an individual-participant meta-analysis evaluating both guided and self-guided ICBT for depression [12]. In addition, client engagement with self-guided ICBT and other self-guided DMHIs tends to be low [13-15], particularly in real-world observational research, where completion rates were found in one systematic review to range from 0.5% to 28.6% [14]. However, self-guided ICBT and other self-guided DMHIs can be implemented on a large scale with minimal human or financial inputs required [7,16], making them cost-effective [17] and—many have argued—justifiable despite their tendency to be less effective than therapist-guided DMHIs or face-to-face psychotherapies [16,18].

There appears to be a growing consensus that DMHIs can be designed to be more engaging for clients [5,19-21], which may have particular implications for mitigating the problem of low engagement in self-guided DMHIs. The persuasive system design framework [22] describes 28 specific design principles for improving engagement divided into four categories: (1) primary task support principles, which facilitate completion of treatment tasks (eg, tailoring content for specific user groups and presenting complex tasks in a series of simple steps); (2) dialogue support principles, which facilitate dialogue between an intervention and its users (eg, automated praise, reminders, or virtual rewards); (3) system credibility support principles, which help ensure that users perceive interventions as credible (eg, endorsements from credible third parties and inclusion of experts in the design process); and (4) social support principles, which enable users to support each other in their use of an intervention (eg, opportunities for users to support and learn from each other). Systematic reviews and meta-analyses have shown that persuasive design characteristics can predict treatment engagement [20] and symptom change [5], but research assessing the impact of specific persuasive design principles is limited.

Online discussion forums facilitate social support principles of persuasive design. Previous research suggests that they may help support engagement and outcomes in self-guided DMHIs. For example, participants in forum-only control conditions across several studies have demonstrated promising outcomes [23-26], prompting the authors of one paper to conclude that they “could be regarded as an intervention” in and of themselves [23]. In total, 3 randomized trials have shown that self-guided ICBT [27,28] or self-guided bibliotherapy [3] accompanied by an online discussion forum exhibited equivalent outcomes to those of therapist-guided ICBT. Another randomized trial experimentally demonstrated that adding an online discussion forum to guided ICBT improved engagement [29]. Together, these studies suggest that forums could help improve engagement and outcomes in ICBT—potentially bridging the engagement and efficacy gap between guided and self-guided ICBT—but there are no previous randomized trials experimentally evaluating the impact of a forum on engagement and outcomes in self-guided ICBT.

In 2019, a clinical research unit called PSPNET was founded to develop, deliver, and conduct research on free ICBT interventions tailored specifically for Canadian first responders and other public safety personnel (PSP), who frequently experience potentially psychologically traumatic events [30], report high rates of mental health problems [31], and face unique barriers to accessing mental health care (eg, stigma within their workplaces) [32,33]. At the time this study was conducted, PSPNET offered 2 therapist-guided ICBT interventions to Canadian PSP—one transdiagnostic and the other posttraumatic stress disorder (PTSD) specific—both of which have shown promising outcomes with respect to symptom change, engagement, and treatment satisfaction [34-36]. However, at the time of this study, PSPNET was unable to offer guided ICBT services to PSP in all Canadian provinces and territories, highlighting an opportunity to develop a self-guided ICBT intervention that could be delivered with minimal resources required to PSP anywhere in Canada. No previous research has evaluated self-guided ICBT tailored specifically for PSP.

### Objectives and Hypotheses

Broadly speaking, we designed this study to evaluate self-guided ICBT among Canadian PSP while addressing several questions concerning the role of online discussion forums in self-guided ICBT. Specifically, we sought to address the following four objectives:

1. To evaluate transdiagnostic, self-guided ICBT tailored for PSP with respect to treatment engagement, outcomes, and satisfaction.
2. To evaluate whether adding an online discussion forum to transdiagnostic, self-guided ICBT tailored for PSP would improve engagement and outcomes.
3. To evaluate whether participant engagement in the online discussion forum would moderate treatment outcomes.
4. To conduct a mixed methods analysis of participant feedback on the discussion forum.

We hypothesized that participants in both conditions would experience at least small to moderate reductions in symptoms of depression, anxiety, and posttraumatic stress, consistent with recent meta-analytic evidence [5,6]. Second, we hypothesized that participants randomly assigned to receive access to an online discussion forum would show greater engagement and more favorable treatment outcomes than those randomly assigned to receive ICBT without a discussion forum.

## Methods

### Study Design

We used a randomized trial design with 2 conditions: an ICBT plus peer support forum condition and an ICBT-only condition. Participants in both conditions were given free access to a self-guided ICBT program called the *Self-Guided PSP Wellbeing Course*. For participants in the ICBT plus peer support forum condition, but not for those in the ICBT-only condition, this ICBT course included a built-in online discussion forum. Participants were not blinded to their own condition as it is not possible to hide therapy content from those receiving therapy, but the experimental manipulation was described only in general terms (ie, without reference to forums) such that participants were blind to how the condition to which they were assigned differed from the condition to which they were not assigned. We adopted a simple randomization approach [37], which we implemented via a random number generator with a 1:1 ratio. We registered the methodological protocol for this research on ClinicalTrials.gov (ID NCT05145582) and made 2 deviations from it. First, we removed the Sheehan Disability Scale [38] from our planned outcome measures because we were unable to obtain permission to use it. Second, we ultimately carried out our primary quantitative analyses using multilevel modeling (MLM) instead of generalized estimating equations, as we had originally planned, because a paper was published during the course of this research that provided a compelling rationale and detailed recommendations for using MLM in treatment-control pretest-posttest-follow-up study designs [39]. We followed the CONSORT (Consolidated Standards of Reporting Trials) guidelines [40] in reporting the findings of this research. This research was conducted within the context of author HCM’s doctoral dissertation, and we would refer interested readers to this dissertation (expected to be publicly available in or around October 2024) for further details about this research.

### Setting

This study was conducted in Canada, where publicly funded mental health services have not met public demand, leading many Canadians to access private mental health care instead [41]. Canadians have access to DMHIs through various Canadian organizations [42]. There are also thousands of mental health–related phone apps and websites available in Canada and other countries [43], but many of these services are not empirically supported. All research activities pertaining to this study were carried out at the University of Regina in Saskatchewan, Canada.

### Participants, Recruitment, and Enrollment

A power analysis indicated that a minimum of 110 participants would be required to achieve adequate power to detect moderate between-group differences (see Multimedia Appendix 1 [3,5,8,27,34,44-47] for details on our sample size planning). Participants were informed about this research via paid social media advertisements (ie, Twitter [subsequently rebranded X] and Facebook), emails forwarded to PSP by leaders of PSP organizations, presentations to PSP organizations by author HCM and PSPNET’s clinicians, and word of mouth. To be eligible to take part, prospective participants were required to self-report (1) being aged ≥18 years; (2) residing in Canada; (3) working, volunteering, or having previously worked or volunteered as a PSP; (4) being able to access the internet via a computer; and (5) not experiencing significant ongoing concerns related to alcohol or drug use, psychotic symptoms, or manic symptoms.

Prospective participants accessed this study through a web page on PSPNET’s website, which provided information about the study. Upon reviewing a consent form and consenting to participate, they accessed a series of eligibility screening questionnaires through Qualtrics (Qualtrics International Inc). We contacted prospective participants by email to inform them of their eligibility, and eligible participants were asked to confirm their intent to take part in the study, after which they were randomly assigned to 1 of the 2 conditions and provided with a temporary password to access the version of the *Self-Guided* *PSP Wellbeing Course* (ie, with or without the peer support forum) to which they had been assigned. All randomization and enrollment procedures were carried out by author HCM and research assistant Julia Gregory (see the *Acknowledgments* section). Recruitment took place between December 6, 2021, and September 26, 2022.

### Measures

#### Primary Outcome Measures

##### Patient Health Questionnaire–9

The Patient Health Questionnaire–9 (PHQ-9) is a psychometrically sound, 9-item questionnaire assessing depressive symptoms [48,49]. Possible total scores range from 0 to 27, and a score of ≥10 suggests that a respondent’s symptoms are clinically significant [50].

##### Generalized Anxiety Disorder–7

The Generalized Anxiety Disorder–7 (GAD-7) is a 7-item questionnaire assessing generalized anxiety that has demonstrated strong psychometric properties [49,51]. Total scores can range from 0 to 21, with a score of ≥10 suggesting clinically significant symptoms [49,51].

##### PTSD Checklist for DSM-5

PTSD Checklist for DSM-5 (PCL-5) is a psychometrically sound questionnaire assessing PTSD symptoms [52]. Responses to its 20 items sum to a total score ranging from 0 to 80, and a score of ≥33 indicates that a respondent likely meets criteria for a PTSD diagnosis [53,54].

#### Secondary Outcome Measures

##### Brief Resilience Scale

The Brief Resilience Scale (BRS) is a 6-item questionnaire measure of resilience that has shown good psychometric properties [55,56]. Each item has 5 response options with associated numerical values ranging from 1 to 5, and a higher mean score across items indicates greater resilience.

##### Flourishing Scale

The Flourishing Scale (FS) is an 8-item questionnaire assessing flourishing across various domains of life (eg, relationships, meaning and purpose, and feeling of competence). It has demonstrated good psychometric properties [57,58]. Total scores can range from 8 to 56, with greater scores indicating a greater degree of flourishing.

##### Treatment Satisfaction Questionnaire

We administered a bespoke questionnaire designed to assess treatment satisfaction and solicit feedback on the *Self-Guided PSP Wellbeing Course* through a mix of yes or no, Likert-scale, and open-ended text response items. For participants in the ICBT plus peer support forum condition, the Treatment Satisfaction Questionnaire included several additional items pertaining to the forum. Open-ended questions about both the course and the forum were designed to solicit both positive and constructive feedback.

##### Adapted Session Rating Scale

We administered a modified version of the Session Rating Scale (SRS), a 4-item questionnaire originally designed to assess client perspectives on the quality of the therapeutic alliance in face-to-face therapy [59]. It has good psychometric qualities [60,61]. Items are rated on a 7-point Likert scale from 0 (*absolutely disagree*) to 6 (*absolutely agree*) and assess the therapeutic bond, goal agreement, task agreement, and overall alliance quality. Following an approach taken in another study [62], we adapted the SRS to measure patient-program alliance.

##### Program Use Questionnaire

We administered a brief bespoke questionnaire assessing engagement with the *Self-Guided PSP Wellbeing Course* and, if applicable, the peer support forum. Specifically, this questionnaire was designed to assess effort put into the course; the perceived helpfulness of the course; and, if applicable, use and perceived helpfulness of the peer support forum. Program use patterns were also assessed via automatic collection of program use data (eg, number of lessons and additional resources accessed).

##### Health Service Use Questionnaire

We also administered a bespoke questionnaire to assess use of health care services for mental health challenges during eligibility screening and at 8 and 20 weeks after enrollment. In the interest of brevity, and because this questionnaire is peripheral to the primary objectives of this study, we do not describe the outcomes of this questionnaire in this paper.

#### Pre-Enrollment Measures

During eligibility screening, we administered a bespoke participant information questionnaire assessing demographic, occupational, and clinical characteristics; an ICBT feedback questionnaire assessing pre-enrollment knowledge and attitudes toward ICBT; the Credibility/Expectancy Questionnaire (CEQ) [63]; the Alcohol Use Disorders Identification Test [64]; and the Drug Use Disorders Identification Test [65].

#### Administration of Measures

During eligibility screening, we administered the PHQ-9, GAD-7, PCL-5, BRS, FS, participant information questionnaire, ICBT feedback questionnaire, CEQ, Alcohol Use Disorders Identification Test, and Drug Use Disorders Identification Test. At 2, 4, 6, and 8 weeks after enrollment, we administered the Program Use Questionnaire. At 8 weeks after enrollment, we also administered the PHQ-9, GAD-7, PCL-5, BRS, FS, and Treatment Satisfaction Questionnaire. At 20 weeks after enrollment, we administered the PHQ-9, GAD-7, PCL-5, BRS, and FS. Our research team encouraged participants to complete the questionnaires via emails and phone calls but did not urge participants to use the *Self-Guided PSP Wellbeing Course*.

### Intervention

The *Self-Guided* *PSP Wellbeing Course* is an 8-week self-guided, transdiagnostic ICBT program that can be accessed through a web browser. It includes 5 core lessons, each consisting of a welcome video, a series of slides with instructive text and diagrams, an audio file covering the same clinical content as the lesson slides, illustrative case stories, frequently asked questions, downloadable homework activities called “DIY Guides,” and quotes from previous clients. These lessons included an introduction to the cognitive behavioral model and psychoeducation to help participants recognize and understand their symptoms (lesson 1); skills to help participants recognize and challenge unhelpful thoughts (lesson 2); skills for managing physiological underarousal and overarousal symptoms (lesson 3); skills for managing behavioral symptoms (lesson 4); and strategies for maintaining treatment gains, setting goals, and preventing future relapses (lesson 5). The course also included 14 additional resources covering a wide range of topics (eg, assertiveness, physical pain, and intimate relationships) and automated email reminders to encourage engagement.

The *Self-Guided* *PSP Wellbeing Course* is effectively a self-guided version of a previously developed therapist-guided ICBT course called the *PSP Wellbeing Course* [34,36]; aside from the provision of therapist guidance in the latter but not the former, the courses are practically identical. PSPNET developed the *PSP Wellbeing Course* by tailoring an existing ICBT program called the *Wellbeing Course* to meet the needs of Canadian PSP based on feedback provided by Canadian PSP in a series of interviews, focus groups, and questionnaires [33,66]. The original *Wellbeing Course* was initially developed by the eCentreClinic at Macquarie University, Australia, and has since shown excellent outcomes among the general population in Australia [67] and Canada [68].

### Discussion Forum

The peer support forum was built into the *Self-Guided PSP Wellbeing Course*. It included 11 sections (eg, one for each of the 5 lessons, one for discussing families and relationships, and one for discussing workplace issues). It was monitored daily and moderated as required each business day by author HCM, who posed questions to spark discussion and responded to participants’ posts.

### Ethical Considerations

This study was approved by the University of Regina Research Ethics Board (file 2021-130). Before taking part, all participants were provided with an informed consent form, which described the following: the objectives of the research, the research team, what participation would entail (ie, the intervention and questionnaires), possible risks and benefits of participating, project funding, considerations regarding concurrent mental health treatments, right to withdraw, limits to confidentiality, risks to privacy, precautions to improve security of participant information (both PSPNET’s precautions and precautions that participants could take), uses of participants’ data (ie, eligibility determination and research), information on accessing research results, a statement indicating that participants would not be compensated for taking part, and an invitation to contact our team with any questions or concerns. All participant data were deidentified before analysis. Due to ethical concerns related to the exclusion of individuals reporting suicidal ideation from DMHI research [69], we tried to refer prospective participants reporting suicidal ideation to more intensive services and clarified that the *Self-Guided PSP Wellbeing Course* is not a crisis service, but we allowed them to participate if they met the eligibility criteria.

### Analyses

#### Quantitative Data Analyses

We carried out all quantitative analyses using SPSS (version 28; IBM Corp). We did not statistically test for group differences in pre-enrollment variables as it is not meaningful to test the probability that group differences occurred by chance when it is already known—due to random assignment—that they did [70]. Instead, we inspected the magnitude of group differences and planned to conduct sensitivity analyses to assess the impact of marked differences should we observe any. We compared changes in scores on the PHQ-9, GAD-7, PCL-5, FS, and BRS across conditions using an MLM approach recommended by Sharpe and Cribbie [39]. We used an intention-to-treat approach [71] including all participants in the analyses, and we accounted for missing data using the restricted maximum likelihood estimation method, which previous research suggests is preferable to maximum likelihood estimation for MLM when random effects are included [72]. Each model was run using a random intercept and fixed effects of group, time (as a categorical variable), and the interaction between group and time. We used a variance components covariance structure [73]. We also produced a *G* matrix for each model consistent with the recommendations by Sharpe and Cribbie [39]. We used scatterplots and histograms to test the assumptions of linearity, homoscedasticity of residuals, and normality of residuals [74]. For each of the 5 outcome variables, we conducted one model for the entire sample and one model for the subset of participants with clinically significant scores at the pre-enrollment time point, which we defined using established cutoff scores for the PHQ-9 (≥10), GAD-7 (≥10), or PCL-5 (≥33) and scores in the lower 3 quartiles on the FS (<48) and BRS (<4.0). Therefore, we ran 10 models in total.

In each of the 10 MLM models, we conducted 5 contrasts. In total, 2 contrasts were designed to assess for interactions between group and time—that is, to identify any differences between groups with respect to changes in dependent variables over time—including one contrast for the period between the pre-enrollment time point and 8 weeks after beginning treatment and one for the period between the pre-enrollment time point and 20 weeks after beginning treatment. We collapsed the 2 groups for 3 additional contrasts to determine whether changes in questionnaire scores over time were statistically significant—one contrast for the period between the pre-enrollment time point and 8 weeks after beginning treatment, one for the period between the pre-enrollment time point and 20 weeks after beginning treatment, and one for the period between 8 weeks and 20 weeks after beginning treatment. These latter 3 contrasts were designed to assess whether participants in the *Self-Guided PSP Wellbeing Course* experienced significant changes in their mental health.

Finally, in each of the 10 MLM models, we investigated the effects of five covariates on changes in questionnaire scores over time: (1) the number of lessons that participants accessed, (2) the number of additional resources that participants accessed, (3) CEQ credibility scores, (4) CEQ expectancy scores, and (5) gender. These analyses are not central to the objectives of this study but may be of interest to some readers; accordingly, a rationale for the inclusion of these specific covariates, methods and results pertaining to our covariate analyses, and a brief discussion of the findings of those analyses are shown in Multimedia Appendix 2 [63,75-80].

In addition to the MLM models, we used 2-tailed independent-sample *t* tests and chi-square tests to assess for group differences in treatment satisfaction and program use. Upon observing possible group differences in rates of questionnaire completion, we conducted additional (non-prespecified) chi-square tests to evaluate their significance.

#### Qualitative Data Analyses

We conducted qualitative analyses using a content analysis approach to explore participant feedback on the peer support forum and the *Self-Guided PSP Wellbeing Course* in general [81]. After removing identifying information from the data, author HCM identified categories using a descriptive, inductive approach and grouped those categories into overarching themes. Given the relatively small amount of data, this was carried out using an Excel (Microsoft Corp) spreadsheet. The initial codebook was refined through meeting with author HDH and Dr Janine Beahm (see the *Acknowledgments* section).

It is a common practice for researchers using qualitative methods to engage in reflexivity, which is a practice of reflection on how the researchers’ positionality might affect the process or outcomes of qualitative research [82]. Being neither PSP nor ICBT clients, the authors do not identify as members of the population under study, potentially granting the authors a degree of neutrality in describing participants’ reported experiences but also potentially impeding their ability to fully understand those experiences [82]. In addition, the authors held certain attitudes and beliefs (eg, that ICBT can be helpful for many people and that forums may be able to enhance ICBT) that may have influenced the process and outcomes of this research. Nevertheless, we endeavored to minimize the risk of bias in this research by (1) including neutrally worded questions to solicit both positive and negative feedback; (2) conducting content analysis as descriptively as possible and avoiding even minor inferences and assumptions; (3) separating qualitative data from other data that could cause bias in coding (eg, demographic and clinical characteristics) before analysis; and (4) involving 3 researchers, as noted previously, in checking the accuracy of our coding.

## Results

### Participants

**Figure 1:**
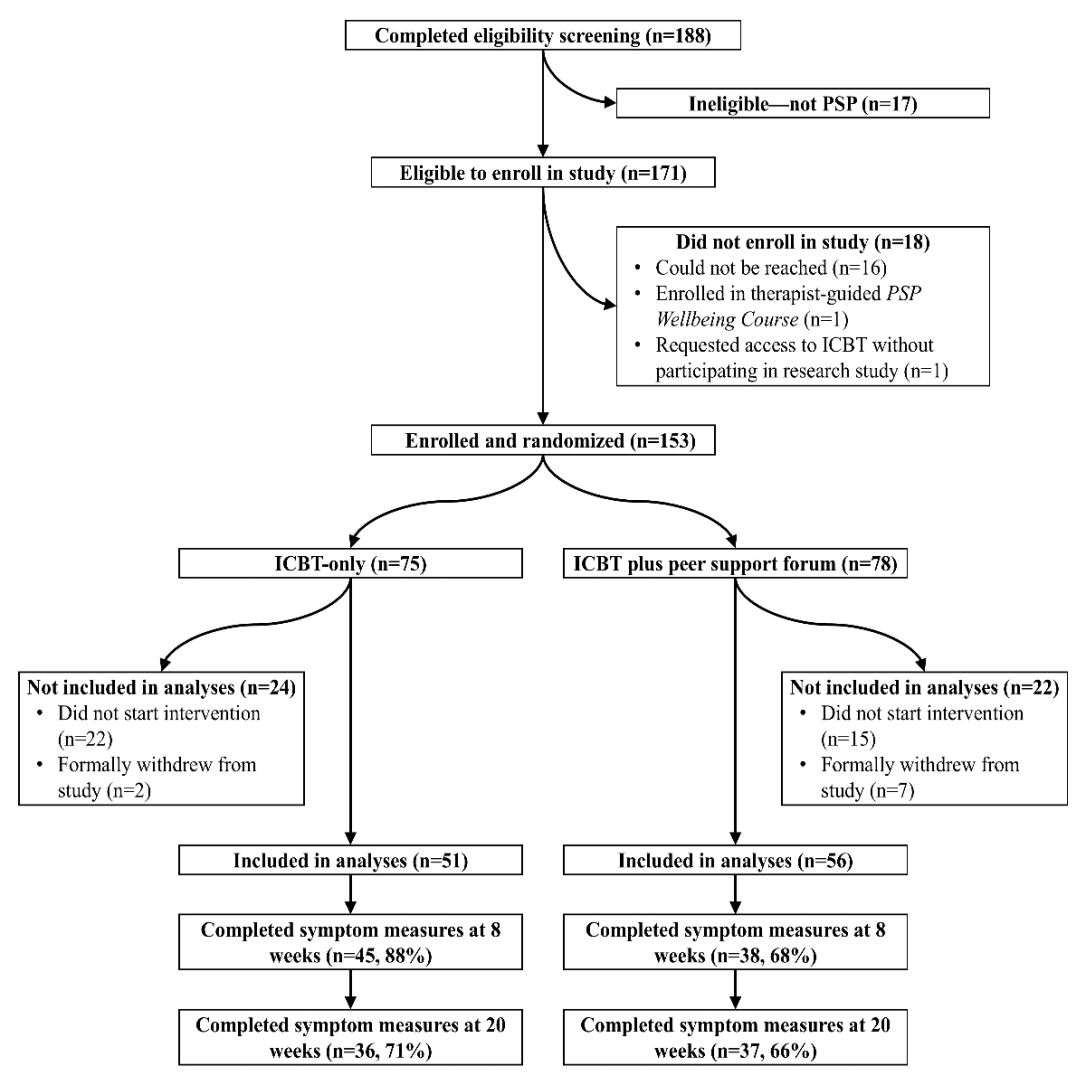
Flowchart displaying enrollment, program use, and questionnaire completion. ICBT: Internet-delivered cognitive behavioral therapy; PSP: public safety personnel.

Of the 188 prospective participants who completed the web-based screening, 153 (81.4%) were enrolled in the study and randomized, and 107 (56.9%) were included in our analyses. A flowchart displaying enrollment, program use, and questionnaire completion is shown in Figure 1. Of note, Figure 1 shows that 36 participants in the ICBT-only condition completed symptom measures at 20 weeks after enrollment; one of these participants completed the PHQ-9 and GAD-7 but not the PCL-5. Participant characteristics are shown in Table 1. Chi-square tests evidenced that the difference between conditions with respect to the proportion of participants who completed posttreatment questionnaires was statistically significant at 8 weeks (n=107, *χ*^2^_1_=6.4, *P*=.01) but not at 20 weeks (n=107, *χ*^2^_1_=0.5, *P*=.47).

**Table 1:** Characteristics of participating public safety personnel (PSP; n=107).

Characteristics		All participants	ICBT^a^-only condition (n=51)	ICBT plus peer support forum condition (n=56)
**Gender, n** **(%)**				
	Women	62 (57.9)	28 (54.9)	34 (60.7)
	Men	45 (42.1)	23 (45.1)	22 (39.3)
**Marital status, n (%)**				
	Married, common-law marriage, or living with a partner	78 (72.9)	38 (74.5)	40 (71.4)
	Not married, in a common-law marriage, or living with a partner	29 (27.1)	13 (25.5)	16 (28.6)
**Parental status, n (%)**				
	Has ≥1 children	70 (65.4)	36 (70.6)	34 (60.7)
	Has no children	37 (34.6)	15 (29.4)	22 (39.3)
**Province or territory, n** **(%)**				
	British Columbia	23 (21.5)	13 (25.5)	10 (17.9)
	Ontario	21 (19.6)	10 (19.6)	11 (19.6)
	Alberta	15 (14)	9 (17.6)	6 (10.7)
	New Brunswick	12 (11.2)	4 (7.8)	8 (14.3)
	Nova Scotia	11 (10.3)	4 (7.8)	7 (12.5)
	Prince Edward Island	10 (9.3)	5 (9.8)	5 (8.9)
	Saskatchewan	8 (7.5)	4 (7.8)	4 (7.1)
	Manitoba	4 (3.7)	0 (0)	4 (7.1)
	Newfoundland and Labrador	1 (0.9)	1 (2)	0 (0)
	Northwest Territories	1 (0.9)	1 (2)	0 (0)
	Quebec	1 (0.9)	0 (0)	1 (1.8)
**Community size, n** **(%)**				
	<100,000 residents	72 (67.3)	32 (62.7)	40 (71.4)
	≥100,000 residents	35 (32.7)	19 (37.3)	16 (28.6)
**Level of education, n** **(%)**				
	No university degree	56 (52.3)	26 (51)	30 (53.6)
	University degree	51 (47.7)	25 (49)	26 (46.4)
**Years of experience as PSP^b^, n (%)**				
	≥10	75 (70.1)	37 (72.5)	38 (67.9)
	0-9	32 (29.9)	14 (27.5)	18 (32.1)
**PSP occupational area, n** **(%)**				
	Police	37 (34.6)	19 (37.3)	18 (32.1)
	Corrections	23 (21.5)	10 (19.6)	13 (23.2)
	Paramedics or related emergency service	16 (15)	9 (17.6)	7 (12.5)
	Fire	11 (10.3)	5 (9.8)	6 (10.7)
	Communications (eg, 911 dispatch)	7 (6.5)	2 (3.9)	5 (8.9)
	Other	13 (12.1)	6 (11.8)	7 (12.5)
**Ethnicity, n** **(%)**				
	Indigenous (ie, First Nations, Inuit, or Metis)	7 (6.5)	6 (11.8)	1 (1.8)
	White	96 (89.7)	42 (82.4)	54 (96.4)
	Other ethnic minority group	3 (2.8)	2 (3.9)	1 (1.8)
	Prefer not to answer	1 (0.9)	1 (2)	0 (0)
**Age group (y), n** **(%)**				
	20-29	7 (6.5)	4 (7.8)	3 (5.4)
	30-39	27 (25.2)	16 (31.4)	11 (19.6)
	40-49	47 (43.9)	22 (43.1)	25 (44.6)
	50-59	21 (19.6)	9 (17.6)	12 (21.4)
	60-69	5 (4.7)	0 (0)	5 (8.9)
Age (y), mean (SD)		44.50 (9.28)	42.97 (8.94)	45.90 (9.45)

^a^ICBT: internet-delivered cognitive behavioral therapy.

^b^PSP: public safety personnel.

### Changes in Questionnaire Scores

We observed a common pattern of results across all 10 MLM models: (1) all statistical assumptions were met; (2) we did not identify statistically significant effects of group or group-by-time interactions (*P*≥.17 in all cases); (3) contrasts showed no effect of group on score change at 8 or 20 weeks for any measure; (4) there was a statistically significant and favorable effect of time (ie, scores on the PHQ-9, GAD-7, and PCL-5 decreased and scores on the FS and BRS increased over time); (5) contrasts showed statistically significant improvement in scores at 8 and 20 weeks for all measures; and (6) we identified residual variance, suggesting that models were likely missing predictor variables that could have helped account for estimates of dependent variables (which was expected given that covariates were tested separately via contrasts rather than being included in MLM models).

There were also some differences across MLM models. Certain contrasts for the PHQ-9 and PCL-5 showed further improvement in symptoms from 8 to 20 weeks. Further details of MLM results are reported in Table 2 (estimated means and percentage changes) and Table 3 (contrasts). Unaltered questionnaire scores observed among respondents at each time point are shown in Multimedia Appendix 3 [83-86].

**Table 2:** Summary of estimated means of scores on questionnaires and percentage changes over time.

Questionnaires and time points		Entire sample				Clinical subsamples		
		Both conditions	ICBT^a^ only	ICBT plus peer support forum	Both conditions		ICBT only	ICBT plus peer support forum
**PHQ-9^b,c^**								
	Pre-enrollment time point, estimated mean	9.51	9.69	9.34	14.21		14.35	14.07
	8 weeks, estimated mean (% change from pre-enrollment time point)	7.26 (–23.7)	6.96 (–28.2)	7.53 (–19.4)	9.58 (–32.6)		9.86 (–31.3)	9.31 (–33.9)
	20 weeks, estimated mean (% change from pre-enrollment time point)	6.01 (–36.7)	5.94 (–38.7)	6.08 (–34.9)	7.94 (–44.1)		7.96 (–44.5)	7.93 (–43.6)
**GAD-7^d,e^**								
	Pre-enrollment time point, estimated mean	8.10	8.34	7.88	13.79		14.47	13.15
	8 weeks, estimated mean (% change from pre-enrollment time point)	6.19 (–23.5)	5.92 (–29.0)	6.44 (–18.3)	8.71 (–36.9)		9.08 (–37.3)	8.35 (–36.5)
	20 weeks, estimated mean (% change from pre-enrollment time point)	5.31 (–34.5)	5.47 (–34.4)	5.16 (–34.4)	8.25 (–40.2)		8.46 (–41.6)	8.05 (–38.8)
**PCL-5^f,g^**								
	Pre-enrollment time point, estimated mean	26.86	24.73	28.80	46.64		47.47	46.08
	8 weeks, estimated mean (% change from pre-enrollment time point)	18.71 (–30.3)	17.61 (–28.8)	19.71 (–31.6)	28.68 (–38.5)		33.84 (–28.7)	25.17 (–45.4)
	20 weeks, estimated mean (% change from pre-enrollment time point)	16.49 (–38.6)	15.52 (–37.2)	17.37 (–39.6)	23.67 (–49.3)		24.78 (–47.8)	22.91 (–50.3)
**FS^h,i^**								
	Pre-enrollment time point, estimated mean	40.83	40.92	40.75	37.63		37.16	38.02
	8 weeks, estimated mean (% change from pre-enrollment time point)	42.22 (3.4)	41.86 (2.3)	42.55 (4.4)	39.78 (5.7)		38.49 (3.6)	40.86 (7.5)
	20 weeks, estimated mean (% change from pre-enrollment time point)	43.35 (6.2)	43.28 (5.8)	43.41 (6.5)	41.11 (9.3)		40.34 (8.6)	41.76 (9.8)
**BRS^j,k^**								
	Pre-enrollment time point, estimated mean	3.28	3.33	3.24	2.92		2.96	2.88
	8 weeks, estimated mean (% change from pre-enrollment time point)	3.51 (6.8)	3.47 (4.0)	3.54 (9.3)	3.29 (12.9)		3.32 (12.3)	3.27 (13.5)
	20 weeks, estimated mean (% change from pre-enrollment time point)	3.48 (5.9)	3.55 (6.5)	3.41 (5.3)	3.26 (11.8)		3.33 (12.5)	3.20 (11.0)

^a^ICBT: internet-delivered cognitive behavioral therapy.

^b^PHQ-9: Patient Health Questionnaire–9.

^c^Entire sample—both conditions: n=107, ICBT-only condition: n=51, and ICBT plus peer support forum: n=56; clinical subsamples—both conditions: n=53, ICBT-only condition: n=26, and ICBT plus peer support forum: n=27.

^d^GAD-7: Generalized Anxiety Disorder–7.

^e^Entire sample—both conditions: n=107, ICBT-only condition: n=51, and ICBT plus peer support forum: n=56; clinical subsamples—both conditions: n=39, ICBT-only condition: n=19, and ICBT plus peer support forum: n=20.

^f^PCL-5: PTSD Checklist for DSM-5.

^g^Entire sample—both conditions: n=107, ICBT-only condition: n=51, and ICBT plus peer support forum: n=56; clinical subsamples—both conditions: n=42, ICBT-only condition: n=17, and ICBT plus peer support forum: n=25.

^h^FS: Flourishing Scale.

^i^Entire sample—both conditions: n=107, ICBT-only condition: n=51, and ICBT plus peer support forum: n=56; clinical subsamples—both conditions: n=81, ICBT-only condition: n=37, and ICBT plus peer support forum: n=44.

^j^BRS: Brief Resilience Scale.

^k^Entire sample—both conditions: n=107, ICBT-only condition: n=51, and ICBT plus peer support forum: n=56; clinical subsamples—both conditions: n=78, ICBT-only condition: n=37, and ICBT plus peer support forum: n=41.

**Table 3:** Summary of contrasts assessing changes in questionnaire scores over time.

Variables		Entire sample				Clinical subsamples		
		*t* test (*df*)	*P* value	Cohen *d*	*t* test (*df*)		*P* value	Cohen *d*
**PHQ-9^a,b^**								
	Pre-enrollment time point to 8 weeks after beginning treatment	–4.44 (158.78)	<.001	–0.70	–6.80 (76.52)		<.001	–1.55
	Pre-enrollment time point to 20 weeks after beginning treatment	–6.55 (160.38)	<.001	–1.03	–8.39 (78.87)		<.001	–1.89
	8-20 weeks after beginning treatment	–2.20 (156.57)	.03	–0.35	–2.11 (77.13)		.04	–0.48
**GAD-7^c,d^**								
	Pre-enrollment time point to 8 weeks after beginning treatment	–4.18 (158.10)	<.001	–0.66	–6.67 (54.53)		<.001	–1.81
	Pre-enrollment time point to 20 weeks after beginning treatment	–5.77 (159.88)	<.001	–0.91	–6.56 (56.78)		<.001	–1.74
	8-20 weeks after beginning treatment	–1.71 (155.65)	.09	–0.27	–0.52 (54.08)		.61	–0.14
**PCL-5^e,f^**								
	Pre-enrollment time point to 8 weeks after beginning treatment	–5.67 (151.91)	<.001	–0.92	–8.39 (57.98)		<.001	–2.20
	Pre-enrollment time point to 20 weeks after beginning treatment	–6.88 (152.70)	<.001	–1.11	–10.58 (58.75)		<.001	–2.76
	8-20 weeks after beginning treatment	–1.42 (149.50)	.16	–0.23	–2.51 (56.12)		.02	–0.67
**FS^g,h^**								
	Pre-enrollment time point to 8 weeks after beginning treatment	2.10 (159.08)	.04	0.33	2.61 (118.39)		.01	0.48
	Pre-enrollment time point to 20 weeks after beginning treatment	3.67 (159.97)	<.001	0.58	4.11 (119.64)		<.001	0.75
	8-20 weeks after beginning treatment	1.60 (157.12)	.11	0.26	1.55 (117.03)		.12	0.29
**BRS^i,j^**								
	Pre-enrollment time point to 8 weeks after beginning treatment	3.00 (160.80)	.003	0.47	4.94 (115.02)		<.001	0.92
	Pre-enrollment time point to 20 weeks after beginning treatment	2.55 (162.06)	.01	0.40	4.23 (116.22)		<.001	0.78
	8-20 weeks after beginning treatment	–0.29 (158.65)	.77	–0.05	–0.39 (112.77)		.70	–0.07

^a^PHQ-9: Patient Health Questionnaire–9.

^b^Entire sample: n=107; clinical subsamples: n=53.

^c^GAD-7: Generalized Anxiety Disorder–7.

^d^Entire sample: n=107; clinical subsamples: n=39.

^e^PCL-5: PTSD Checklist for DSM-5.

^f^Entire sample: n=107; clinical subsamples: n=42.

^g^FS: Flourishing Scale.

^h^Entire sample: n=107; clinical subsamples: n=81.

^i^BRS: Brief Resilience Scale.

^j^Entire sample: n=107; clinical subsamples: n=78.

### Program Use

There was no statistically significant difference between groups with respect to the number of lessons participants accessed by 8 weeks (t_105_=–0.28; *P=*.78; Cohen *d*=–0.05) or 20 weeks (t_105_=0.82; *P=*.42; Cohen *d*=0.16). Collapsing across groups, a sizeable minority of participants accessed all 5 lessons of the *Self-Guided PSP Wellbeing Course* by 8 weeks (30/107, 28%) or 20 weeks (46/107, 43%). Nearly half (48/107, 44.9%) accessed 4 of 5 lessons by 8 weeks, whereas more than half (59/107, 55.1%) accessed 4 of 5 lessons by 20 weeks. Participants accessed an average of 3.33 (SD 5.00) additional resources. Responses to the Program Use Questionnaire collapsed across groups and averaged across time points (ie, 2, 4, 6, and 8 weeks) showed that participants most commonly reported putting “some effort” into the course (39%), followed by “a little effort” (30.9%), “no effort” (17.5%), and “a lot of effort” (12.5%), with no participants reporting “a great deal of effort” at any time point.

### Treatment Satisfaction

The Treatment Satisfaction Questionnaire and SRS were completed by 68% (38/56) of the participants in the ICBT plus peer support forum condition and 88% (45/51) of the participants in the ICBT-only condition. Two-tailed independent-sample *t* tests and chi-square tests showed no statistically significant differences between groups with respect to any treatment satisfaction variables (*P*≥.47 in all cases). Accordingly, we present the results collapsed across groups in Table 4.

We qualitatively analyzed responses to open-ended questions from 61.7% (66/107) of the participants, which we organized into 3 main themes: positive feedback, negative or constructive feedback, and comments about personal circumstances or preferences that do not reflect the perceived helpfulness of the *Self-Guided PSP Wellbeing Course*. The results are shown in Table 5.

**Table 4:** Descriptive statistics on treatment satisfaction collapsed across conditions.

Variables		Values
**Was it worth your time doing this course? (n=82), n (%)**		
	Yes	79 (96)
	No	3 (4)
**Would you feel confident recommending this treatment to a friend? (n=83), n (%)**		
	Yes	78 (94)
	No	5 (6)
**Overall, how satisfied were you with the treatment? (n=83)**		
	Very dissatisfied (0), n (%)	0 (0)
	Dissatisfied (1), n (%)	0 (0)
	Neutral (2), n (%)	31 (37)
	Satisfied (3), n (%)	45 (54)
	Very satisfied (4), n (%)	7 (8)
	Values, mean (SD)	2.71 (0.62)
**How has participating in this course affected your confidence that you can learn to manage your symptoms? (n=82)**		
	Greatly reduced (0), n (%)	3 (4)
	Reduced (1), n (%)	3 (4)
	No change (2), n (%)	21 (26)
	Increased (3), n (%)	49 (60)
	Greatly increased (4), n (%)	6 (7)
	Values, mean (SD)	2.63 (0.82)
**I would have preferred to communicate with a therapist by email while working through the PSP Wellbeing Course (n=82), n (%)**		
	Strongly disagree	2 (2)
	Disagree	14 (17)
	Neutral	32 (39)
	Agree	27 (33)
	Strongly agree	7 (9)
**I would have preferred to communicate with a therapist by phone while working through the PSP Wellbeing Course (n=82), n (%)**		
	Strongly disagree	3 (4)
	Disagree	19 (23)
	Neutral	32 (39)
	Agree	20 (24)
	Strongly agree	8 (10)
**Adapted SRS^a^, mean (SD)**		
	Bond (n=83)	4.55 (1.13)
	Goals (n=82)	4.67 (1.01)
	Tasks (n=82)	4.37 (1.50)
	Overall (n=82)	4.55 (1.29)

^a^SRS: Session Rating Scale.

**Table 5:** Results of content analysis of feedback on the Self-Guided PSP Wellbeing Course (n=66).

Theme, subtheme, and category			Example quote	Frequency, n (%)
**Positive feedback**				
	**Clinical content**			
		Positive feedback on stories or case examples	“I liked the stories cause it helped me relate and see other people are having these experiences.” [Participant 1801]	18 (27)
		Positive feedback on DIY^a^ guides	“DIY Guides are very informative and easy to understand.” [Participant 1256]	17 (26)
		Positive feedback on additional resources	“I liked the resource library to be accessed for follow up and reminders.” [Participant 1757]	13 (20)
		Positive feedback on course content (eg, thorough, understandable, and relatable)	“It goes into explanations that in person therapy doesn’t seem to have time for, or, that in person therapists don’t think to cover.” [Participant 1392]	8 (12)
		Positive feedback on lessons	“Lessons were straight forward and easy to comprehend.” [Participant 1583]	5 (8)
		Positive feedback on tools and skills taught in the course	“Gave me a framework to understand what has been affecting me and how to work on it productively. I have taken my time, more than intended by the course I think, to practice the skills.” [Participant 1175]	6 (9)
		Course acted as a helpful reminder of previously learned skills and information	“Good refresher and reminder of important concepts.” [Participant 1342]	4 (6)
	**Format and delivery**			
		Liked that the course was self-guided, self-paced, or accessible at any time and location	“[Liked] being able to work on the course on my own timeline, when I was in the right headspace. It didn’t feel forced.” [Participant 1091]	12 (18)
		Liked the format or structure of the course or the presentation or delivery of information	“I liked how the course was structured.” [Participant 1648]	10 (15)
		Liked being able to download or print course materials or review them again in the future	“It is nice to have the resources to go back to in the future.” [Participant 1648]	5 (8)
		Liked the reminder emails	“[Liked] reminders to keep at it.” [Participant 1225]	3 (5)
	**Other positive feedback**			
		General statement of liking the course	“[Liked] honestly, all of it.” [Participant 1092]	2 (3)
		No positive feedback provided	—^b^	2 (3)
**Constructive or negative feedback**				
	**Clinical content**			
		Disliked the stories, did not find them helpful, or provided feedback on them	“I didn’t find the stories particularly helpful.” [Participant 1154]	5 (8)
		Course was too basic or recommendation for a second course with more tools	“I thought it would be longer and more in depth.” [Participant 1742]	3 (5)
		Some content seemed redundant or unnecessary	“I found the lessons and DIY guides a bit repetitive (they covered a lot of the same material).” [Participant 1173]	2 (3)
		Other suggestions for improving clinical content	“I was approaching this as a preventative course as opposed to a treatment course so I found that the examples were not something I identified with. It would be wonderful if there was a separate course for individuals looking to build skills to help prevent a slide into negative mental health.” [Participant 1258]	3 (5)
	**Format and delivery**			
		Difficulty or dislike concerning the current use of timelines and reminders to motivate completion	“I needed more time and felt somewhat anxious when the reminders were coming about a new section and I was behind.” [Participant 1503]	7 (11)
		Would prefer if the course included therapist support	“I wished I also had the therapist to help keep me on track and discuss some of my thoughts and feelings that came up while taking the course.” [Participant 1801]	5 (8)
		Disliked amount of reading or suggested more video content	“[Disliked] a lot of reading. Hard to stay focused.” [Participant 1546]	3 (5)
		Course was not mobile friendly	“The slides were difficult to read in a phone. Sitting at a computer isn’t always an option for privacy.” [Participant 1181]	1 (2)
	**Other constructive or negative feedback**			
		No dislikes identified or constructive feedback provided	“There is nothing I didn’t like.” [Participant 1242]	29 (44)
**Comments about personal circumstances or preferences**				
	Limited time, energy, or capacity to work on the course or unexpected life circumstances posing a barrier to progression in the course		“Nothing you can do but life threw me a curve the past couple weeks, very sick kitten so that was my immediate concern and this fell to the wayside.” [Participant 1816]	6 (9)
	Hard time with web-based courses in general, preference for in-person courses or would benefit more from in-person courses		“I would benefit more from in-person treatment, but am reluctant to participate.” [Participant 1225]	3 (5)

^a^DIY: do-it-yourself.

^b^Not applicable.

### Online Discussion Forum Use and Satisfaction

Only 9% (5/56) of the participants in the ICBT plus peer support forum condition posted in the forum, creating 9 posts in total. The moderator (author HCM) created an additional 16 posts in an effort to spark discussion. The Treatment Satisfaction Questionnaire was completed by 38 participants in the ICBT plus peer support forum condition, 14 (37%) of whom reported that they did not use the forum. Of the remaining 24 participants, 1 (4%) reported feeling “very satisfied” with the forum overall, 11 (46%) reported feeling “satisfied,” 8 (33%) reported feeling “neutral,” 4 (17%) reported feeling “dissatisfied,” and none reported feeling “very dissatisfied.” Among the 38 participants in the ICBT plus peer support forum who completed the Treatment Satisfaction Questionnaire were 15 (39%) who reported reading between “a few” posts and “all or nearly all” posts, including participants who reported that reading others’ posts was “highly beneficial” (n=2, 13%), “beneficial” (n=3, 20%), “somewhat beneficial” (n=6, 40%), and “not beneficial at all” (n=4, 27%).

We received meaningful, analyzable feedback on the peer support forum from 52% (29/56) of the participants, identifying 17 categories of feedback, which we grouped into 3 general themes: positive feedback, constructive or negative feedback, and other personal reactions to the forum. The results of this content analysis are shown in Table 6.

**Table 6:** Results of content analysis of feedback on the peer support forum (n=29).

Theme, subtheme, and category			Example quote		Frequency, n (%)
**Positive feedback**					
	Liked that the forum was supportive, open, or free of judgment	“Judgement-free, supportive space to use when/if/how helpful.” [Participant 1217]		2 (7)	
	Liked reading others’ comments or seeing a variety of perspectives	“[Liked] variety of viewpoints.” [Participant 1217]		2 (7)	
	Liked not feeling alone	“It’s nice to know you’re not alone.” [Participant 1816]		2 (7)	
	Liked that the forum was an option	“[Liked] that it was an option.” [Participant 1978]		1 (3)	
	Did not like anything about the forum	“I did not [like the forum]. Possibly I didn’t connect properly?” [Participant 1721]		2 (7)	
**Constructive or negative feedback**					
	Dislike, discomfort, or difficulty opening up to or being vulnerable with others	“[Did not post because] police culture does not encourage sharing or vulnerability with mental health. Peer forums are not a tool we are comfortable with. Especially with the association of privacy and information release in our jobs.” [Participant 1181]		8 (28)	
	Disliked the low level of forum activity or did not post due to low activity level	“[Disliked that] it was not an active forum and often nothing had been posted.” [Participant 1978]		7 (24)	
	Unaware of forum or comment that more prompts would result in greater forum use	“[Did not post because the forum] wasn’t emphasized enough as an available tool or resource during the course. I also did not know it was available to me.” [Participant 1095]		4 (14)	
	Would prefer a scheduled live chat to asynchronous posts	“Wished it was more of a real time chat.” [Participant 1801]		2 (7)	
	General statement of dislike for or disinterest in the forum	“I did not like it.” [Participant 1130]		2 (7)	
	Too much involvement from the moderator	“[Disliked that the forum was]...very monitored?” [Participant 1721]		1 (3)	
	Disliked nothing about the forum	“[Disliked] nothing.” [Participant 1241]		1 (3)	
**Other personal reactions to the forum**					
	Did not post because of other demands or not enough time	“[Did not post because] work and life demands paused my participation in the program.” [Participant 1584]		4 (14)	
	Participant did not feel that they had anything of value to add to forum	[Did not post because] “I felt I didn’t have anything to add of value.” [Participant 1816]		3 (10)	
	Comment on how it felt to post on the forum	“[It felt] very difficult, vulnerable to do, felt unburdened/heard after posting.” [Participant 1217]		2 (7)	
	Misconception that participants cannot respond to each other	“[Disliked that the forum] seemed like a question and answer type without being able to respond to each other.” [Participant 1721]		1 (3)	
	Did not feel a need to post because other aspects of the course were sufficient	“[Did not post because] I am still stuck on capturing my thoughts and found that the FAQ suffices.” [Participant 1168]		1 (3)	

## Discussion

### Principal Findings

ICBT is an effective mental health treatment [1], but clients demonstrate somewhat less favorable clinical outcomes [5-12] and engagement [13-15] when it is offered in a purely self-guided format. Persuasive design principles represent a possible means of improving engagement and outcomes in DMHIs [5,20], and preliminary evidence supports the use of social support principles of persuasive design implemented via online discussion forums [3,23-29], but there is a dearth of experimental research directly evaluating the impact of forums in ICBT or other DMHIs. Research has also shown that Canadian PSP benefit considerably from tailored, guided ICBT [34-36], but previous research has not evaluated self-guided ICBT among Canadian PSP. We conducted a randomized trial to assess the impact of adding an online discussion forum to self-guided ICBT and evaluate outcomes of tailored, self-guided ICBT among Canadian PSP.

Participants showed large improvements in symptoms of depression, anxiety, and posttraumatic stress, which supported and surpassed our hypothesis of at least small to moderate reductions in symptoms. Most meta-analyses of self-guided DMHIs have not reported pretest-posttest effect sizes, but our results for changes in depression over time compare favorably to the pretest-posttest effect size of *d*=0.78 reported in one meta-analysis of self-guided DMHIs for depression [87]. Changes in flourishing and resilience were more modest, potentially because the *Self-Guided PSP Wellbeing Course* was designed to reduce symptoms of mental disorders but not explicitly designed to improve flourishing or resilience. Nevertheless, the finding of improvements in flourishing and resilience makes an important contribution to the research literature as research on the effects of ICBT on these constructs is scarce.

The symptom change and treatment satisfaction demonstrated in this study were roughly comparable to those observed in research on the guided version of the *PSP Wellbeing Course*, likely because the 2 courses included practically identical treatment materials. However, engagement with the guided version was markedly better, with only 5.7% of enrolled PSP failing to access or withdrawing from the intervention (compared to 46/153, 30% in this study), 76.1% of participants accessing at least 4 of the 5 lessons of the course within 8 weeks (compared to 48/107, 44.9% in this study), and 57.3% completing the course within 8 weeks (compared to 30/107, 28% in this study) [36]. These findings suggest that therapists play a pivotal role in both initiating and sustaining Canadian PSP’s engagement in ICBT. This conclusion aligns with those of previous research showing that PSP very frequently cite therapist guidance as a liked aspect of ICBT [88] and with a broader research literature showing that engagement tends to be lower in self-guided than in guided DMHIs [13-15]. Nevertheless, engagement with the *Self-Guided PSP Wellbeing Course* in this study appears to compare favorably to that of other research on self-guided DMHIs. In a systematic review, Kelders et al [20] found, on average, a 54.2% rate of “intended use” (ie, some engagement but not necessarily completion) of internet interventions for mental health, including both guided and self-guided interventions. Another systematic review found real-world completion rates ranging from 0.5% to 28.6% for self-guided DMHIs [14]. Engagement in the present study may have been enhanced by the structure provided by the randomized trial design as this kind of study design was found to predict greater engagement in the review by Kelders et al [20]. Interestingly, only a minority of participants in the present study indicated that they would have preferred to receive therapist guidance via email or phone while taking the course.

The results failed to support our hypothesis that participants assigned to the ICBT plus peer support forum condition would demonstrate greater engagement and treatment outcomes. Given participants’ limited engagement with the peer support forum, this was unsurprising. The proportion of participants who posted in the forum (5/56, 9%) was far lower than proportions of 53% [26] and 50.6% [28] reported in previous studies of forums in DMHIs. Similarly, the mean number of posts per participant (0.16) was far lower than the means of 13.1 [89], 4.5 [89], 2.2 [29], and 1.86 [28] reported in previous studies. We are aware of only one previous study in which a lower proportion (ie, 7%) of participants posted in a forum accompanying a DMHI [90]. Despite low engagement with the peer support forum, some participants reported feeling satisfied with it and indicated that reading posts was beneficial, with qualitative feedback suggesting that some participants felt that the forum was supportive and helped them feel that they were not alone.

There are several possible reasons for the low engagement with the forum in this study. First, qualitative feedback suggested that many participants felt uncomfortable opening up to others and showing vulnerability, with one participant explicitly attributing this to “police culture,” suggesting that forums may be a poor fit for many PSP. Second, the fact that the *Self-Guided PSP Wellbeing Course* was transdiagnostic, with different participants experiencing different symptoms, may have led participants to feel that they did not have much in common with other forum users and, therefore, may also have detracted from their comfort in sharing their experiences. Third, forums may be particularly helpful as an adjunct to treatment for certain conditions; indeed, much of the past research supporting the use of forums in ICBT has been conducted within the context of ICBT for social anxiety [3,23,27,28]. Fourth, the PSP who self-selected into this study may have been particularly interested in independently accessing a self-guided treatment, whereas PSP who were interested in sharing their experiences with others may have opted for other mental health care options (including PSPNET’s therapist-guided ICBT for PSP in provinces where it was available). Finally, there was likely room for improvement with respect to the structure and implementation of the forum and our efforts to encourage participants to use it.

There was only one statistically significant difference observed between conditions: a greater proportion of participants completed questionnaires at 8 weeks in the ICBT-only condition. It remains unclear why this occurred. It could be a spurious finding. It could also be that participants in the ICBT plus peer support forum condition inferred from the minimal forum engagement that engagement with the study *as a whole* was low and were less likely to complete questionnaires due to the phenomenon of social normative influence [91].

### Strengths, Limitations, and Future Research

This study benefitted from a mixed methods approach, allowing for both a quantitative evaluation of treatment outcomes and a qualitative exploration of participants’ experiences. Another strength of this study was its ecological validity as we evaluated the *Self-Guided PSP Wellbeing Course* and the peer support forum under the conditions in which they were designed to be implemented. This study also had important limitations. We did not include a control condition with which to compare outcomes of the course, and our inclusion of multiple outcome measures in separate analyses increased our familywise error rate. We expect that every discussion forum is unique and its social dynamics are unpredictable; therefore, a key limitation of this study is that it is, in a sense, a case study of a single forum with results that may not generalize well to other forums. This study was also sufficiently powered to detect only moderate differences between conditions, and due to an unexpectedly high rate of withdrawal from the study or failure to begin the intervention after we had ceased recruitment, we ultimately included 3 fewer participants in our analyses than originally planned. Finally, because we did not exclude participants with minimal or mild pre-enrollment symptoms from this research, floor effects are likely present in quantitative analyses conducted among our entire sample.

Future research can expand on this study and address the limitations noted previously in several ways. Although the peer support forum in this study had no demonstrable effect on treatment outcomes, previous research has shown excellent outcomes for online discussion forums [3,23-29], highlighting a need for further experimental research to evaluate the impact of forums on treatment outcomes in self-guided DMHIs. We are aware of a large factorial randomized trial assessing, among other things, the impact of an online discussion forum on treatment outcomes in ICBT, but the results of this trial are not yet available [92]. It would also be helpful for future research to identify common characteristics of forums that function well and those that do not, further explore DMHI users’ perspectives on forums, and identify strategies for improving engagement with forums drawing on the persuasive system design framework and other work. With respect to outcomes of self-guided ICBT tailored for PSP, future research could compare treatment outcomes against a control condition, assess longer-term outcomes, and assess additional outcomes that we did not assess.

### Conclusions

ICBT has shown excellent outcomes for treating a range of psychological concerns among PSP [34-36] and the general population [1]. Self-guided ICBT is more scalable but shows poorer engagement and outcomes than therapist-guided ICBT [5-15]. There is emerging evidence suggesting that persuasive design may help improve engagement and outcomes in ICBT [5,19-21], but further research is needed. We conducted a randomized trial, finding that transdiagnostic self-guided ICBT tailored specifically for PSP showed good outcomes, but PSP randomly assigned to receive access to a built-in online discussion forum showed limited engagement with it and no evidence of benefitting from it. Our findings support the continued implementation of self-guided ICBT. Our findings contrast with those of previous research on discussion forums in DMHIs, which have generally shown promising engagement and outcomes [3,23-29,89], highlighting a need for more research to clarify the circumstances under which forums may help improve engagement and outcomes in DMHIs. More broadly, as DMHIs become increasingly popular, there is a great need for more research identifying possible strategies to make them more engaging and effective, including—but not limited to—further research evaluating the impact of specific persuasive design principles.

## Appendix

Multimedia Appendix 1 Sample size planning.

Multimedia Appendix 2 Covariate analyses.

Multimedia Appendix 3 Observed descriptive statistics on questionnaire scores.

## Abbreviations

BRS: Brief Resilience Scale
CEQ: Credibility/Expectancy Questionnaire
CONSORT: Consolidated Standards of Reporting Trials
DMHI: digital mental health intervention
FS: Flourishing Scale
GAD-7: Generalized Anxiety Disorder–7
ICBT: internet-delivered cognitive behavioral therapy
MLM: multilevel modeling
PCL-5: PTSD Checklist for DSM-5
PHQ-9: Patient Health Questionnaire–9
PSP: public safety personnel
PTSD: posttraumatic stress disorder
SRS: Session Rating Scale

## Data Availability

The data sets generated and analyzed during this study are not publicly available due our commitment to our participants to securely store their data and refrain from sharing them with anyone outside of our research team.
