# Supplementary material for: Impact of an Online Discussion Forum on Self-Guided Internet-Delivered Cognitive Behavioral Therapy for Public Safety Personnel: Randomized Trial
Source: J Med Internet Res. 2024 Aug 14;26:e59699. doi: 10.2196/59699 (PMC11358668; doi:10.2196/59699)
Supplement: Multimedia Appendix 1 [file jmir_v26i1e59699_app1.docx]

*Multimedia Appendix 1: Sample Size Planning*

**Overview of Sample Size Planning**

The purpose of this appendix is to describe our power analysis and other considerations related to sample size planning. We used the “longpower” package in R [44] to estimate a required sample size. Drawing on initial Patient Health Questionnaire-9 (PHQ-9) results in the therapist-guided version of the *PSP Wellbeing Course* [34], we estimated a baseline variance of 30.5 and a correlation between measurements of *r* = .58. To our knowledge, prior research had not experimentally assessed the impact of an online discussion forum on symptom change in ICBT. However, we arrived at an estimated effect size of *g* = 0.49 by averaging estimated effect sizes from two speculative lines of reasoning, which are described in the next section of this appendix. Assuming a power level of 80% and a significance level of .05, our power analysis showed that we would require 55 participants per condition (i.e., 110 in total).

We anticipated that not all participants would complete post-treatment measures, but this did not warrant any changes to our estimated sample size because we accounted for missing data in our analyses. We did account for 3.5% of our participants withdrawing from the study or not beginning the *Self-Guided PSP Wellbeing Course* after enrolling, based on initial results from the therapist-guided version of the *PSP Wellbeing Course* [34]. We therefore concluded that we would require a sample of 114 participants. This estimate was calculated as a minimum acceptable sample size, as described in our registered trial protocol (ClinicalTrials.gov ID NCT05145582), and we planned to recruit more participants if possible to increase our statistical power and buffer against the possibility of losing more than the estimated 3.5% of our sample to withdrawal from the study or non-initiation of the course.

**Details of Effect Size Estimates**

As described above, our estimated effect size of *g* = 0.49 was based on two speculative lines of reasoning. First, a self-guided ICBT intervention for social anxiety, which included an online discussion forum, showed a within-groups effect size of *d* = 1.54 [27]. In a later study, the same research group found a within-groups effect size of *d* = 1.01 collapsed across transdiagnostic and disorder-specific self-guided ICBT interventions for social anxiety that were similar to the ICBT intervention in the first study in look, feel, and content but did not include online discussion forums [45]. There were no differences in effectiveness between the transdiagnostic and disorder-specific ICBT programs in the second study; therefore, even though the reported effect size was collapsed across the transdiagnostic and disorder-specific ICBT programs, it was approximately reflective of the effectiveness of the disorder-specific ICBT program, which was virtually identical to the program used in the first study [27]). The samples in these two studies were relatively similar with regard to demographic characteristics, suggesting that sample characteristics are unlikely to account for much of the difference in effect size observed between these two studies. Given that the ICBT programs for social anxiety appear to be very similar to each other in most respects aside from the inclusion of a forum in the first study, we speculated that the forum might be the primary factor accounting for the observed difference in effect sizes. Therefore, we estimated that we might find a similar difference in within-groups effect sizes (i.e., a difference of *g* = 0.53) favouring participants assigned to the ICBT + Peer Support Forum condition relative to the ICBT-Only condition. Assuming similar standard deviations of symptom scores across conditions (which was expected due to random assignment), any difference between groups with respect to within-groups effect sizes could be expected to translate into a similarly sized difference in between-groups effect sizes (i.e., of approximately *g* = 0.53).

Our second line of reasoning to help estimate an effect size for our power analysis was drawn from research showing no differences in effectiveness between guided ICBT and self-guided ICBT supplemented by online discussion forums [27,46]. Another study found no difference in effectiveness between self-guided bibliotherapy and guided ICBT when both were supplemented by an online discussion forum [3]. Because these three studies suggest that online discussion forums can bridge the effectiveness gap between guided ICBT and self-guided ICBT/bibliotherapy, we estimated an effect size of forums by quantifying the effectiveness gap between guided and self-guided ICBT. To do so, we reviewed a recent meta-analysis of self-guided ICBT for symptoms of depressive and anxiety disorders, which revealed mean effect sizes of *g* = 0.22 for symptoms of depressive disorders and *g* = 0.45 for symptoms of anxiety disorders compared to control conditions [5]. We also reviewed recent meta-analyses of guided ICBT for symptoms of depressive and anxiety disorders. Two meta-analyses of guided ICBT and/or computerized cognitive behavioural therapy (CCBT) found identical mean effect sizes of *g* = 0.67 for depressive disorders [8,47]. A meta-analysis of CCBT and ICBT for anxiety disorders evidenced mean effect sizes of *g* = 0.70 for symptoms of generalized anxiety disorder, *g* = 0.92 for symptoms of social anxiety disorder, and *g* = 1.31 for symptoms of panic disorder [47]. We averaged available effect sizes, weighting each by the total number of participants included across all trials from which they were derived, to find a weighted mean effect size of *g* = 0.91 for guided ICBT for symptoms of anxiety disorders. Therefore, we estimated the effectiveness gap between guided and self-guided interventions to be *g* = 0.45 for depressive disorders (0.67 - 0.22 = 0.45) and *g* = 0.46 for anxiety disorders (0.91 - 0.45 = 0.46), which we averaged to *g* = 0.455.

Taking the average of *g* = 0.53 (from our first line of reasoning) and *g* = 0.455 (from our second line of reasoning), we arrived at a final estimated effect size of *g* = 0.49. Incidentally, this estimated effect size is identical to a within-groups effect size of *d* = 0.49 found among participants assigned to a discussion forum control condition in a prior randomized trial of an ICBT program, lending some additional support to the notion that forums could result in effect sizes of this approximate magnitude. We appreciate that our effect size estimate has methodological limitations. Nevertheless, in the absence of solid prior evidence upon which to base an effect size estimate, we believe that our reasoning, though highly speculative, represents a favourable alternative to choosing an estimated effect size without any reasoning.
