# Supplementary material for: Impact of an Online Discussion Forum on Self-Guided Internet-Delivered Cognitive Behavioral Therapy for Public Safety Personnel: Randomized Trial
Source: J Med Internet Res. 2024 Aug 14;26:e59699. doi: 10.2196/59699 (PMC11358668; doi:10.2196/59699)
Supplement: Multimedia Appendix 2 [file jmir_v26i1e59699_app2.docx]

*Multimedia Appendix 2: Covariate Analyses*

**Overview**

In each of our multilevel modeling (MLM) models, we investigated the effects of five covariates on treatment outcomes: (a) the number of lessons participants accessed by 20 weeks post-enrollment; (b) the number of additional resources participants accessed by 20 weeks post-enrollment; (c) participants’ scores on the credibility subscale of the Credibility/Expectancy Questionnaire (CEQ) [63], participants’ scores on the expectancy subscale of the CEQ [63], and participants’ self-identified gender. The purpose of this appendix is to provide a rationale for our selection of these specific covariates, the methods through which we evaluated their impact on outcomes, the results of our covariate analyses, and a brief discussion of those results.

**Rationale for Selected Covariates**

Prior empirical research supports the inclusion of the covariates we analyzed. Research on other versions of the *Wellbeing Course* shows that greater program engagement predicts greater symptom change [75], supporting our inclusion of lessons accessed and additional resources accessed as covariates. Research has also shown that more favourable treatment expectancies predict more favourable treatment outcomes and engagement [76–78], which supported our inclusion of the two CEQ subscales as predictors of outcomes. Finally, one systematic review found that women generally have greater adherence to ICBT than men [76], but a more recent systematic review found that gender is a commonly evaluated but inconsistent predictor of treatment outcomes in internet interventions [79], emphasizing a need for continued research on the possible relationship between gender and treatment outcomes in ICBT and other DMHIs—and therefore supporting our exploratory inclusion of gender as a covariate.

**Methods**

As detailed in the body of our paper, we conducted 10 MLM analyses: one for the entire sample and one for the clinical subsample for each of our 5 outcome measures. For each of the five covariates, we then created a separate version of each of these MLM models that included, as a fixed effect, the interaction term between the covariate and time. In doing so, we were able to assess whether each covariate was a statistically significant predictor of changes in the dependent variable over time. Of note, we had originally planned to examine whether engagement with the online discussion forum would predict changes in dependent variables over time. However, due to minimal engagement with the forum, we omitted forum engagement from our covariate analyses.

**Results**

***Overview***

Gender by time interactions were statistically significant in five of the 10 MLMs, and in each of these cases, *t*-tests showed no statistically significant gender differences at pretreatment, a statistically significant difference at 8 weeks such that men demonstrated more favorable scores at that time, and no statistically significant differences at 20 weeks. The interaction term between time and participants’ expected improvement in functioning on the CEQ (administered at pretreatment) was a statistically significant predictor in the MLMs of FS scores for both the entire sample and the lower-three-quartiles subsample. In both cases, greater expected improvement in functioning was associated with statistically significantly greater flourishing at eight and 20 weeks post-enrollment and slightly (but not statistically significantly) greater flourishing at pretreatment. Finally, the interaction between time and perceived treatment credibility on the CEQ was a statistically significant predictor of BRS scores within the entire sample (but not the clinical subsample), such that participants who perceived the *Self-Guided PSP Wellbeing Course* as more credible at pretreatment showed less resilience at 20 weeks post-enrollment. Lessons accessed and Additional Resources accessed did not covary with changes on any dependent variable, either within the entire sample or within clinical subsamples for each dependent variable. Detailed results are presented for gender and the two CEQ subscales below.

***Gender***

First, there was a statistically significant gender by time interaction in predicting PHQ-9 scores among the entire sample, *F*(3, 133.71) = 4.40, *p* = .005. Specifically, men’s PHQ-9 scores were 1.59 points (*SE* = 1.04, 95% CI [-3.64, 0.46]) lower than women’s at pretreatment, *t*(159.18) = -1.53, *p* = .13, Cohen’s *d* = -0.24; 3.99 points (*SE* = 1.12, 95% CI [-6.19, -1.79]) lower at 8 weeks, *t*(187.14) = -3.57, *p* < .001, Cohen’s *d* = -0.52; and 2.27 points (*SE* = 1.16, 95% CI [-4.56, 0.01]) lower at 20 weeks, *t*(202.48) = -1.96, *p* = .051, Cohen’s *d* = 0.28. Second, the interaction term between gender and time predicted PHQ-9 scores among the clinical subsample, *F*(3, 65.43) = 5.65, *p* = .002. Specifically, men’s PHQ-9 scores were 0.83 points (*SE* = 1.22, 95% CI [-3.25, 1.60]) lower than women’s at pretreatment, *t*(83.97) = -0.68, *p* = .50, Cohen’s *d* = -0.15; 5.03 points (*SE* = 1.30, 95% CI [-7.61, -2.44]) lower at 8 weeks, *t*(94.64) = -3.86, *p* < .001, Cohen’s *d* = -0.79; and 2.24 points (*SE* = 1.43, 95% CI [-5.08, 0.59]) lower at 20 weeks, *t*(108.68) = -1.57, *p* = .12, Cohen’s *d* = -0.30. Third, the time by gender interaction term predicted GAD-7 scores among the entire sample, *F*(3, 133.35) = 2.99, *p* = .03. Men’s GAD-7 scores were 1.13 points (*SE* = 0.94, 95% CI [-2.99, 0.73]) lower than women’s at pretreatment, *t*(160.87) = -1.20, *p* = .23, Cohen’s *d* = -0.19; 2.93 points (*SE* = 1.01, 95% CI [-4.92, -0.94]) lower at 8 weeks, *t*(187.32) = -2.90, *p* = .004, Cohen’s *d* = -0.42; and 1.92 points (*SE* = 1.05, 95% CI [-3.99, 0.15]) lower at 20 weeks, *t*(202.59) = -1.83, *p* = .07, Cohen’s *d* = -0.26. Fourth, there was a statistically significant time by gender interaction in predicting GAD-7 scores among the clinical subsample, *F*(3, 49.98) = 3.69, *p* = .02, such that men’s GAD-7 scores were 0.74 points (*SE* = 1.22, 95% CI [-1.70, 3.19]) higher than women’s at pretreatment, *t*(64.07) = 0.61, *p* = .55, Cohen’s *d* = 0.15; 3.44 points (*SE* = 1.37, 95% CI [-6.17, -0.71]) lower at 8 weeks, *t*(72.81) = -2.51, *p* = .01, Cohen’s *d* = -0.59; and 2.64 points (*SE* = 1.57, 95% CI [-5.76, 0.49]) lower at 20 weeks, *t*(79.81) = -1.68, *p* = .10, Cohen’s *d* = -0.38. Finally, the interaction term between time and gender predicted FS scores among the entire sample, *F*(3, 101.93) = 2.74, *p* = .047. Men’s FS scores were 0.56 points (*SE* = 1.68, 95% CI [-2.76, 3.88]) higher than women’s at pretreatment, *t*(115.89) = 0.33, *p* = .74, Cohen’s *d* = 0.06; 4.36 points (*SE* = 1.81, 95% CI [0.79, 7.94]) higher at 8 weeks, *t*(138.08) = 2.41, *p* = .02, Cohen’s *d* = 0.41; and 2.75 points (*SE* = 1.88, 95% CI [-0.96, 6.47]) higher at 20 weeks, *t*(149.24) = 1.46, *p* = .15, Cohen’s *d* = 0.24.

***CEQ Scores***

The interaction term between time and participants’ expected improvement in functioning on the CEQ predicted FS scores among the entire sample, *F*(3, 132.05) = 2.83, *p* = .04. For each percentage of expected improvement in functioning, a participant’s predicted FS score increased by 0.04 points (*SE* = 0.04, 95% CI [-0.03, 0.11]) at pretreatment, *t*(136.76) = 1.19, *p* = .24, Cohen’s *d* = 0.20; 0.08 points (*SE* = 0.04, 95% CI [0.00, 0.15]) at 8 weeks, *t*(157.84) = 2.11, *p* = .04, Cohen’s *d* = 0.34; and 0.10 points (*SE* = 0.04, 95% CI [0.03, 0.18]) at 20 weeks, *t*(176.78) = 2.64, *p* = .009, Cohen’s *d* = 0.40. The same effect was found among participants who had FS scores in the lower three quartiles (i.e., <48) at pretreatment; the interaction between time and expected improvement in functioning predicted FS scores, *F*(3, 100.42) = 3.80, *p* = .01, such that for each percentage point of expected improvement in functioning, a participant’s predicted FS score increased by 0.06 points (*SE* = 0.04, 95% CI [-0.01, 0.13]) at pretreatment, *t*(117.59) = 1.58, *p* = .12, Cohen’s *d* = 0.29; 0.11 points (*SE* = 0.04, 95% CI [0.03, 0.19]) at 8 weeks, *t*(137.48) = 2.73, *p* = .007, Cohen’s *d* = 0.47; and 0.13 points (*SE* = 0.04, 95% CI [0.04, 0.21]) at 20 weeks, *t*(155.69) = 3.02, *p* = .003, Cohen’s *d* = 0.48. Finally, the interaction term between time and perceived treatment credibility scores on the CEQ predicted BRS scores, *F*(3, 128.24) = 3.13, *p* = .03, such that for each point on the CEQ credibility subscale (i.e., the mean response to the first three items of the CEQ, with a possible range from 1 to 9), a participant’s predicted BRS score decreased by 0.10 points (*SE* = 0.06, 95% CI [-0.21, 0.02]), *t*(143.76) = -1.68, *p* = .09, Cohen’s *d* = -0.28 at pretreatment; by 0.11 points (*SE* = 0.06, 95% CI [-0.24, 0.01]), *t*(165.87) = -1.86, *p* = .06, Cohen’s *d* = -0.29 at 8 weeks; and by 0.19 points (*SE* = 0.06, 95% CI [-0.31, -0.07]), *t*(173.70) = -3.05, *p* = .003, Cohen’s *d* = -0.46 at 20 weeks.

**Comments**

In both entire-sample and subsample analyses of changes in depression and anxiety, as well as the entire-sample analysis of changes in flourishing, men showed better outcomes at 8 weeks. These differences were no longer statistically significant at 20 weeks, indicating that men may have experienced *faster* outcomes rather than *better* outcomes per se. However, descriptively, men experienced greater improvement in outcomes than women at 20 weeks, with most comparisons at 20 weeks approaching statistical significance. These results add to a literature of mixed findings concerning the impact of gender on ICBT outcomes [79].

Greater expected improvement in outcomes predicted greater improvement in flourishing. This is consistent with our hypothesis that more favorable treatment expectancies would predict better outcomes, but it should be noted that more favorable treatment expectancies did not predict changes over time in most dependent variables. Also, it is possible that the relationship between positive treatment expectancies and greater improvement in flourishing could be explained by another factor we did not measure, such as optimism. Interestingly, participants who perceived the *Self-Guided PSP Wellbeing Course* to be more credible tended to experience less favorable changes in resilience over the course of the study. It is not obvious why this was the case. One possibility is that skepticism regarding the credibility of the course was related to critical thinking skills, which may have helped participants benefit more from the course. It is also possible this was simply a spurious finding.

Contrary to our hypotheses and previous findings from other versions of the *Wellbeing Course* [75], greater program engagement—measured via lessons accessed and additional resources accessed—did not predict more favorable outcomes on any measure. The absence of the dose-response relationship we expected to find does not appear to be attributable to limited variability in program use (i.e., which would hinder the usefulness of program use variables in predicting any other variable), as there was considerable variability in both lessons accessed and additional resources accessed. One explanation for the finding that engagement did not predict outcomes is that our measures of program use were not an adequate proxy for the degree to which participants successfully learned and applied treatment principles in their lives. It is also worth noting that research on face-to-face psychotherapy has shown that other factors (e.g., extratherapeutic factors, placebo) account for more variability in treatment outcomes than specific models and techniques of treatment [80], suggesting that much of participants’ variability in outcomes likely had little to do with their engagement with the course.
