# Supplementary material for: Impact of an Online Discussion Forum on Self-Guided Internet-Delivered Cognitive Behavioral Therapy for Public Safety Personnel: Randomized Trial
Source: J Med Internet Res. 2024 Aug 14;26:e59699. doi: 10.2196/59699 (PMC11358668; doi:10.2196/59699)
Supplement: Multimedia Appendix 3 [file jmir_v26i1e59699_app3.docx]

*Multimedia Appendix 3: Observed Descriptive Statistics on Questionnaire Scores*

This appendix is supplementary to our formal quantitative analyses. It shows unaltered, observed descriptive statistics for the PHQ-9, GAD-7, PCL-5, FS, and BRS. Table S1 shows descriptive statistics including all available data at each timepoint for these five questionnaires. Table S2 shows all available data on pre-treatment-to-8-week and pre-treatment-to-20-week changes in symptoms for participants in the clinical subsamples for each questionnaire; that is, it includes only participants in the clinical subsamples who provided data at 8 and/or 20 weeks. Table S3 shows rates of symptom deterioration on primary outcome measures among participants who completed those measures at pretreatment and one or both other timepoints. Based on prior research, we defined deterioration of symptoms as an increase of at least six points on the PHQ-9 [83], five points on the GAD-7 [84,85], and 10 points on the PCL-5 [86].

**Table S1.** Descriptive statistics on observed questionnaire scores.

| Outcome variable | | All participants (*n* = 107) | ICBT-Only (*n* = 51) | ICBT + Peer Support Forum (*n* = 56) |
| --- | --- | --- | --- | --- |
| PHQ-9 | |  |  |  |
|  | Pretreatment, *M* (*SD*), *n* | 9.50 (5.58), *n* = 107 | 9.69 (5.64), *n* = 51 | 9.34 (5.57), *n* = 56 |
|  | 8 weeks, *M* (*SD*), *n* | 7.00 (5.62), *n* = 83 | 6.87 (5.88), *n* = 45 | 7.16 (5.37), *n* = 38 |
|  | 20 weeks, *M* (*SD*), *n* | 5.44 (4.56), *n* = 73 | 5.36 (4.32), *n* = 36 | 5.51 (4.85), *n* = 37 |
|  | Score of 10+ at pretreatment, *n* (%) | 53/107 (49.5) | 26/51 (51.0) | 27/56 (48.2) |
|  | Score of 10+ at 8 weeks, *n* (%) | 25/83 (30.1) | 11/45 (24.4) | 14/38 (36.8) |
|  | Score of 10+ at 20 weeks, *n* (%) | 15/73 (20.5) | 7/36 (19.4) | 8/37 (21.6) |
| GAD-7 | |  |  |  |
|  | Pretreatment, *M* (*SD*), *n* | 8.12 (5.19), *n* = 106 | 8.40 (5.61), *n* = 50 | 7.88 (4.82), *n* = 56 |
|  | 8 weeks, *M* (*SD*), *n* | 5.96 (4.92), *n* = 83 | 5.62 (4.83), *n* = 45 | 6.37 (5.06), *n* = 38 |
|  | 20 weeks, *M* (*SD*), *n* | 4.95 (3.92), *n* = 73 | 4.92 (3.98), *n* = 36 | 4.97 (3.91), *n* = 37 |
|  | Score of 10+ at pretreatment, *n* (%) | 39/106 (36.8) | 19/50 (38.0) | 20/56 (35.7) |
|  | Score of 10+ at 8 weeks, *n* (%) | 18/83 (21.7) | 10/45 (22.2) | 8/38 (21.1) |
|  | Score of 10+ at 20 weeks, *n* (%) | 8/73 (11.0) | 4/36 (11.1) | 4/37 (10.8) |
| PCL-5 | |  |  |  |
|  | Pretreatment, *M* (*SD*), *n* | 26.83 (19.07), *n* = 105 | 24.57 (18.89), *n* = 49 | 28.80 (19.17), *n* = 56 |
|  | 8 weeks, *M* (*SD*), *n* | 17.65 (15.01), *n* = 83 | 16.76 (15.87), *n* = 45 | 18.71 (14.06), *n* = 38 |
|  | 20 weeks, *M* (*SD*), *n* | 15.39 (13.39), *n* = 72 | 13.69 (11.97), *n* = 35 | 17.00 (14.58), *n* = 37 |
|  | Score of 33+ at pretreatment, *n* (%) | 42/105 (40.0) | 17/49 (34.7) | 25/56 (44.6) |
|  | Score of 33+ at 8 weeks, *n* (%) | 16/83 (19.3) | 9/45 (20.0) | 7/38 (18.4) |
|  | Score of 33+ at 20 weeks, *n* (%) | 12/72 (16.7) | 3/35 (8.6) | 9/37 (24.3) |
| FS | |  |  |  |
|  | Pretreatment, *M* (*SD*), *n* | 40.83 (8.32), *n* = 107 | 40.92 (8.73), *n* = 51 | 40.75 (8.00), *n* = 56 |
|  | 8 weeks, *M* (*SD*), *n* | 42.29 (8.70), *n* = 83 | 41.91 (9.53), *n* = 45 | 42.74 (7.72), *n* = 38 |
|  | 20 weeks, *M* (*SD*), *n* | 44.15 (8.10), *n* = 73 | 44.61 (8.08), *n* = 36 | 43.70 (8.20), *n* = 37 |
| BRS | |  |  |  |
|  | Pretreatment, *M* (*SD*), *n* | 3.28 (0.84), *n* = 107 | 3.33 (0.87), *n* = 51 | 3.24 (0.81), *n* = 56 |
|  | 8 weeks, *M* (*SD*), *n* | 3.51 (0.81), *n* = 83 | 3.50 (0.85), *n* = 45 | 3.53 (0.77), *n* = 38 |
|  | 20 weeks, *M* (*SD*), *n* | 3.49 (0.84), *n* = 73 | 3.63 (0.81), *n* = 36 | 3.35 (0.86), *n* = 37 |

**Table S2.** Observed pre-post changes in questionnaire scores among clinical subsamples.

| Outcome variable | | All participants (*n* = 107) | ICBT-Only (*n* = 51) | ICBT + Peer Support Forum (*n* = 56) |
| --- | --- | --- | --- | --- |
| PHQ-9 pretreatment to 8 weeks* | | *n* = 43 | *n* = 24 | *n* = 19 |
|  | Pretreatment, *M* (*SD*) | 13.72 (2.97) | 14.04 (3.28) | 13.32 (2.56) |
|  | 8 weeks, *M* (*SD*) | 9.40 (5.52) | 9.71 (5.80) | 9.00 (5.28) |
|  | Change score, *M* (*SD*) | -4.33 (4.86) | -4.33 (5.36) | -4.32 (4.28) |
|  | Percentage change, *M* (*SD*) | -31.6% (35.4%) | -30.8% (38.2%) | -32.4% (32.1%) |
|  | Hedges’ *g* (95% CI) | 0.975 (0.53–1.42) | 0.92 (0.32–1.51) | 1.04 (0.36–1.72) |
|  | Score reduced below 10 at 8 weeks, *n* (%) | 24 (55.8) | 15 (62.5) | 9 (47.4) |
|  | Reliable change of at least -6 points, *n* (%) | 19 (44.2) | 10 (41.7) | 9 (47.4) |
|  | Clinically improved ^a^, *n* (%) | 26 (60.5) | 16 (66.7) | 10 (52.6) |
| PHQ-9 pretreatment to 20 weeks* | | *n* = 33 | *n* = 16 | *n* = 17 |
|  | Pretreatment, *M* (*SD*) | 14.00 (3.09) | 14.31 (3.14) | 13.71 (3.12) |
|  | 20 weeks, *M* (*SD*) | 7.55 (4.56) | 7.63 (4.79) | 7.47 (4.47) |
|  | Change score, *M* (*SD*) | -6.45 (4.71) | -6.69 (5.82) | -6.24 (3.54) |
|  | Percentage change, *M* (*SD*) | -46.1% (33.6%) | -46.8% (40.7%) | -45.5% (25.8%) |
|  | Hedges’ *g* (95% CI) | 1.66 (1.10–2.22) | 1.65 (0.85–2.45) | 1.62 (0.84–2.39) |
|  | Score reduced below 10 at 20 weeks, *n* (%) | 21 (63.6) | 10 (62.5) | 11 (64.7) |
|  | Reliable change of at least -6 points, *n* (%) | 17 (51.5) | 8 (50.0) | 9 (52.9) |
|  | Clinically improved ^a^, *n* (%) | 22 (66.7) | 10 (62.5) | 12 (70.6) |
| GAD-7 pretreatment to 8 weeks* | | *n* = 29 | *n* = 15 | *n* = 14 |
|  | Pretreatment, *M* (*SD*) | 13.76 (2.75) | 14.80 (2.76) | 12.64 (2.34) |
|  | 8 weeks, *M* (*SD*) | 8.72 (4.82) | 9.20 (5.27) | 8.21 (4.44) |
|  | Change score, *M* (*SD*) | -5.03 (4.99) | -5.60 (5.23) | -4.43 (4.83) |
|  | Percentage change, *M* (*SD*) | -36.6% (36.3%) | -37.8% (35.3%) | -35.0 (38.2%) |
|  | Hedges’ *g* (95% CI) | 1.28 (0.72–1.85) | 1.33 (0.54–2.12) | 1.25 (0.44–2.06) |
|  | Score reduced below 10 at 8 weeks, *n* (%) | 18 (62.1) | 8 (53.3) | 10 (71.4) |
|  | Reliable change of at least -6 points, *n* (%) | 16 (55.2) | 10 (66.7) | 6 (42.9) |
|  | Clinically improved ^a^, *n* (%) | 21 (72.4) | 11 (73.3) | 10 (71.4) |
| GAD-7 pretreatment to 20 weeks* | | *n* = 22 | *n* = 10 | *n* = 12 |
|  | Pretreatment, *M* (*SD*) | 13.82 (2.54) | 15.20 (2.62) | 12.57 (1.87) |
|  | 20 weeks, *M* (*SD*) | 8.00 (4.04) | 8.50 (4.01) | 7.58 (4.19) |
|  | Change score, *M* (*SD*) | -5.82 (4.19) | -6.70 (4.32) | -5.08 (4.12) |
|  | Percentage change, *M* (*SD*) | -42.1% (30.3%) | -44.1% (28.4%) | -40.4% (32.8%) |
|  | Hedges’ *g* (95% CI) | 1.73 (1.03–2.42) | 1.98 (0.91–3.05) | 1.54 (0.63–2.45) |
|  | Score reduced below 10 at 20 weeks, *n* (%) | 18 (81.8) | 8 (80.0) | 10 (83.3) |
|  | Reliable change of at least -6 points, *n* (%) | 16 (72.7) | 7 (70.0) | 9 (75.0) |
|  | Clinically improved ^a^, *n* (%) | 19 (86.4) | 9 (90.0) | 10 (83.3) |
| PCL-5 pretreatment to 8 weeks* | | *n* = 31 | *n* = 14 | *n* = 17 |
|  | Pretreatment, *M* (*SD*) | 44.68 (8.67) | 46.21 (7.92) | 43.41 (9.28) |
|  | 8 weeks, *M* (*SD*) | 27.65 (13.82) | 33.14 (15.23) | 23.12 (11.02) |
|  | Change score, *M* (*SD*) | -17.03 (12.00) | -13.07 (13.19) | -20.29 (10.19) |
|  | Percentage change, *M* (*SD*) | -38.1% (26.9%) | -28.3% (28.5%) | -46.7% (23.5%) |
|  | Hedges’ *g* (95% CI) | 1.48 (0.92–2.04) | 1.08 (0.28–1.87) | 1.99 (1.17–2.81) |
|  | Score reduced below 10 at 8 weeks, *n* (%) | 19 (61.3) | 6 (42.9) | 13 (76.5) |
|  | Reliable change of at least -6 points, *n* (%) | 22 (71.0) | 8 (57.1) | 14 (82.4) |
|  | Clinically improved ^a^, *n* (%) | 24 (77.4) | 8 (57.1) | 16 (94.1) |
| PCL-5 pretreatment to 20 weeks* | | *n* = 28 | *n* = 11 | *n* = 17 |
|  | Pretreatment, *M* (*SD*) | 45.54 (11.01) | 45.00 (8.25) | 45.88 (12.72) |
|  | 20 weeks, *M* (*SD*) | 22.43 (12.46) | 22.45 (11.89) | 22.41 (13.17) |
|  | Change score, *M* (*SD*) | -23.11 (12.52) | -22.55 (10.23) | -23.47 (14.10) |
|  | Percentage change, *M* (*SD*) | -50.7% (27.5%) | -50.1% (22.7%) | -51.2% (30.7%) |
|  | Hedges’ *g* (95% CI) | 1.97 (1.33–2.60) | 2.20 (1.14–3.26) | 1.81 (1.01–2.61) |
|  | Score reduced below 10 at 20 weeks, *n* (%) | 20 (71.4) | 9 (81.8) | 11 (64.7) |
|  | Reliable change of at least -6 points, *n* (%) | 25 (89.3) | 10 (90.9) | 15 (88.2) |
|  | Clinically improved ^a^, *n* (%) | 25 (89.3) | 10 (90.9) | 15 (88.2) |
| FS pretreatment to 8 weeks* | | *n* = 61 | *n* = 32 | *n* = 29 |
|  | Pretreatment, *M* (*SD*) | 37.56 (6.69) | 36.84 (7.24) | 38.34 (6.07) |
|  | 8 weeks, *M* (*SD*) | 39.59 (8.21) | 38.34 (8.92) | 40.97 (7.25) |
|  | Change score, *M* (*SD*) | 2.03 (6.55) | 1.50 (7.11) | 2.62 (5.94) |
|  | Percentage change, *M* (*SD*) | 5.4% (17.4%) | 4.1% (19.3%) | 6.8% (15.5%) |
|  | Hedges’ *g* (95% CI) | 0.27 (-0.09–0.63) | 0.19 (-0.31–0.68) | 0.39 (-0.13–0.91) |
| FS pretreatment to 20 weeks* | | *n* = 53 | *n* = 24 | *n* = 29 |
|  | Pretreatment, *M* (*SD*) | 38.11 (6.85) | 38.21 (6.41) | 38.03 (7.30) |
|  | 20 weeks, *M* (*SD*) | 41.72 (7.62) | 41.21 (7.54) | 42.14 (7.79) |
|  | Change score, *M* (*SD*) | 3.60 (6.89) | 3.00 (7.02) | 4.10 (6.87) |
|  | Percentage change, *M* (*SD*) | 9.4% (18.1%) | 7.9% (18.4%) | 10.8% (18.1%) |
|  | Hedges’ *g* (95% CI) | 0.50 (0.11–0.89) | 0.43 (-0.14–1.00) | 0.54 (-0.02–1.07) |
| BRS pretreatment to 8 weeks* | | *n* = 60 | *n* = 32 | *n* = 28 |
|  | Pretreatment, *M* (*SD*) | 2.93 (0.67) | 2.99 (0.71) | 2.85 (0.64) |
|  | 8 weeks, *M* (*SD*) | 3.30 (0.74) | 3.34 (0.85) | 3.24 (0.61) |
|  | Change score, *M* (*SD*) | 0.37 (0.60) | 0.35 (0.62) | 0.40 (0.60) |
|  | Percentage change, *M* (*SD*) | 12.6% (20.5%) | 11.7% (20.7%) | 14.0% (21.1%) |
|  | Hedges’ *g* (95% CI) | 0.52 (0.16–0.89) | 0.45 (-0.05–0.94) | 0.62 (0.09–1.16) |
| BRS pretreatment to 20 weeks* | | *n* = 52 | *n* = 25 | *n* = 27 |
|  | Pretreatment, *M* (*SD*) | 2.92 (0.62) | 3.06 (0.63) | 2.79 (0.58) |
|  | 20 weeks, *M* (*SD*) | 3.25 (0.71) | 3.40 (0.72) | 3.12 (0.69) |
|  | Change score, *M* (*SD*) | 0.33 (0.57) | 0.34 (0.62) | 0.33 (0.53) |
|  | Percentage change, *M* (*SD*) | 11.3% (19.5%) | 11.1% (20.3%) | 11.8% (19.0%) |
|  | Hedges’ *g* (95% CI) | 0.50 (0.11–0.89) | 0.50 (-0.06–1.07) | 0.52 (-0.03–1.06) |

*This table presents data only for participants who completed questionnaires at pretreatment and 8 weeks post-enrollment and/or at pretreatment and 20 weeks post-enrollment. For the PHQ-9, GAD-7, and PCL-5, this table also presents data only for participants with pretreatment scores at or above the clinical cutoffs of 10, 10, and 33, respectively. For the FS and BRS, data are omitted for participants with pretreatment scores in the top quartile (48+ on the FS and 4+ on the BRS).

^a^ Clinical improvement for the PHQ-9, GAD-7, and PCL-5 was defined as a reduction in score to below the clinical cut-off (10, 10, and 33, respectively) and/or a reliable reduction in score (-6, -5, and -10, respectively).

**Table S3.** Rates of symptom deterioration (i.e., worsening)

| Outcome variable | | All participants | ICBT-Only | ICBT + Peer Support Forum |
| --- | --- | --- | --- | --- |
| PHQ-9 | |  |  |  |
|  | 8 weeks post-enrolment | 5/83, 6.0% | 2/45, 4.4% | 3/38, 7.9% |
|  | 20 weeks post-enrolment | 2/73, 2.7% | 1/36, 2.8% | 1/37, 2.7% |
| GAD-7 | |  |  |  |
|  | 8 weeks post-enrolment | 6/82, 7.3% | 2/44, 4.5% | 4/38, 10.5% |
|  | 20 weeks post-enrolment | 3/72, 4.2% | 2/35, 5.7% | 1/37, 2.7% |
| PCL-5 | |  |  |  |
|  | 8 weeks post-enrolment | 4/81, 4.9% | 1/43, 2.3% | 3/38, 7.9% |
|  | 20 weeks post-enrolment | 5/71, 7.0% | 3/34, 8.8% | 2/37, 5.4% |
